# EZH2 promotes chemoresistance in colorectal cancer by inhibiting autophagy through NRP1 suppression

**DOI:** 10.1042/BCJ20240607

**Published:** 2025-05-14

**Authors:** Hong Deng, Qin Xu, Qiang Zhang, Chunfeng Liu, Lei Ren

**Affiliations:** 1 Department of General Surgery (Gastrointestinal Surgery), The Affiliated Hospital of Southwest Medical University, Luzhou, Sichuan, China; 2 Department of Respiratory and Critical Care Medicine, The Affiliated Hospital of Southwest Medical University, Luzhou, Sichuan, China; 3 Inflammation and Allergic Research Unit, The Affiliated Hospital of Southwest Medical University, Luzhou, Sichuan, China; 4 Department of Surgery, Klinikum rechts der Isar, Technical University of Munich, School of Medicine, Munich, Germany

**Keywords:** autophagy, chemoresistance, colorectal cancer, EZH2, NRP1

## Abstract

Colorectal cancer (CRC) is characterized by aggressive tumor growth and chemoresistance, with enhancer of zeste homolog 2 (EZH2) serving a pivotal role in these processes. However, the mechanisms by which it drives tumor proliferation and therapeutic resistance through autophagy regulation remain unclear. Here, we demonstrated that EZH2 expression is elevated in CRC tissues and cell lines, correlating with chemoresistance and diagnostic potential (area under the curve = 0.968). *EZH2* knockdown markedly reduced CRC cell proliferation, while its overexpression promoted tumor growth and increased resistance to irinotecan. Mechanistically, EZH2 suppressed autophagy in CRC cells, a process linked to chemosensitivity, by directly regulating LC3bI/II expression. Notably, EZH2 enhanced the neuropilin-1 (NRP1) level by binding to the *NRP1* promoter, thereby promoting tumor proliferation and irinotecan resistance through autophagy inhibition. *NRP1* depletion partially reversed these effects, underscoring the crucial role of the EZH2-NRP1 axis in CRC. Our findings highlight that targeting the EZH2-NRP1 interaction could represent a novel therapeutic approach to overcoming chemoresistance in CRC.

## Introduction

Colorectal cancer (CRC) remains a leading cause of cancer-related morbidity and mortality worldwide [1], driven by its aggressive nature and frequent resistance to chemotherapy [2–4]. Enhancer of zeste homolog 2 (EZH2), a critical component of the polycomb repressive complex 2 (PRC2), serves as a pivotal regulator of gene silencing through its histone methyltransferase activity [5,6]. Dysregulation of EZH2 has been implicated in various malignancies, including CRC, highlighting its role in promoting tumor progression [7], metastasis [8], and chemoresistance [9]. Despite these insights, the precise mechanisms by which EZH2 contributes to the proliferation and chemoresistance of CRC remain incompletely understood.

Several recent studies suggest that EZH2 modulates autophagy [10,11], a crucial cellular degradation process that maintains homeostasis and influences cancer cell survival under stress [12]. Additionally, neuropilin-1 (NRP1), a membrane-bound glycoprotein, has emerged as a critical mediator of cancer progression [13,14] and chemoresistance [15,16]. Evidence indicates that NRP1 contributes to tumor growth by modulating autophagy [17,18] and alternatively, interacts with autophagy to promote tumor progression and the acquisition of chemoresistance [19]. Understanding the connection between EZH2 and NRP1 and how their interaction drives tumor growth and therapeutic resistance through autophagy regulation could provide novel insights for therapeutic strategies.

In this study, we investigated the role of EZH2 in CRC by assessing its expression in tumor tissues and cell lines, evaluating its effects on cell proliferation and chemoresistance, and exploring its influence on autophagy. Furthermore, we analyzed the regulatory interaction between EZH2 and NRP1, elucidating how this axis contributes to the development of irinotecan resistance. Our findings highlight EZH2 and NRP1 as potential therapeutic targets in CRC and propose new strategies for overcoming chemotherapy resistance.

## Results

### *EZH2* expression is elevated in CRC

**Figure 1:**
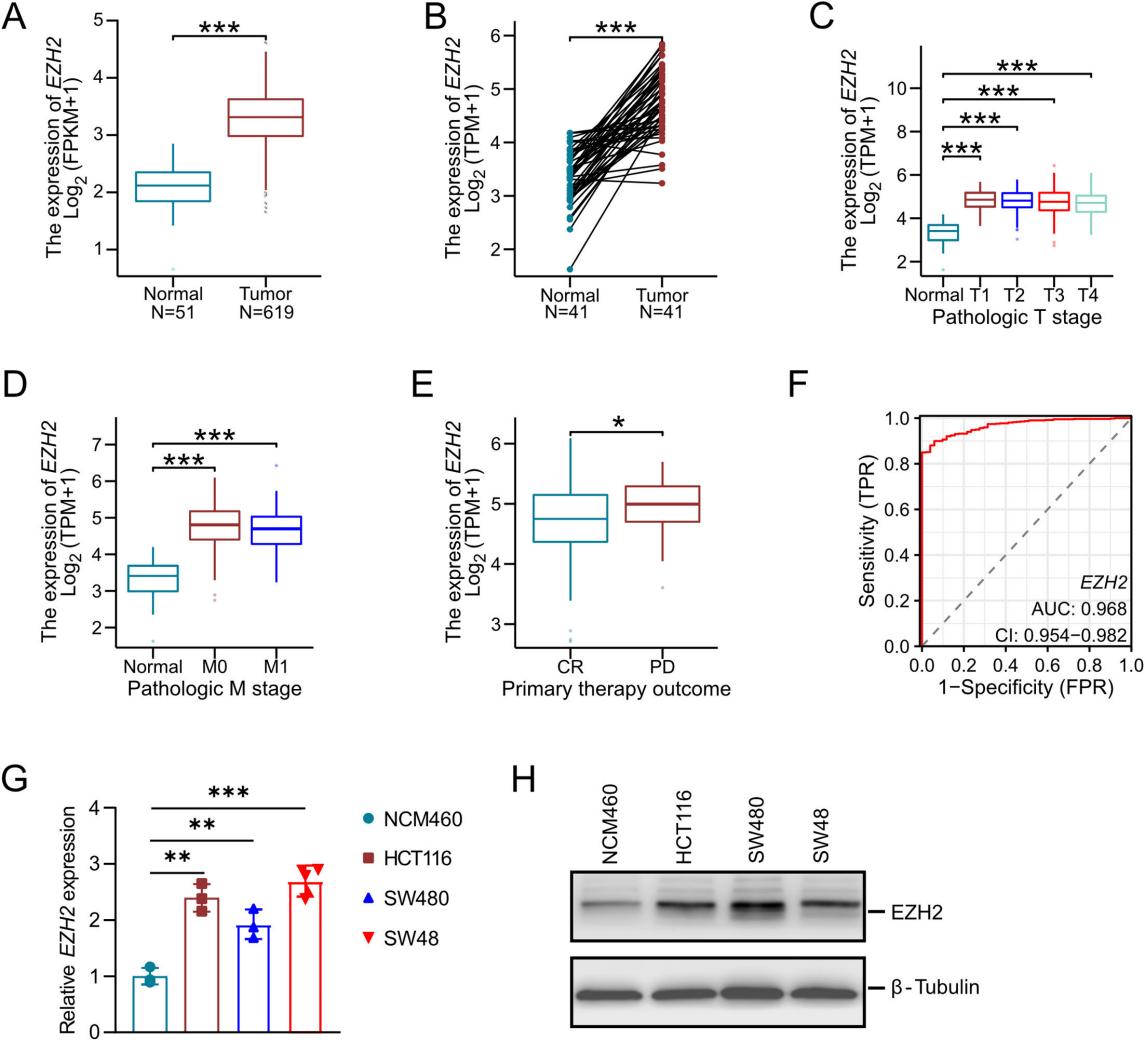
EZH2 expression in CRC tissues and cell lines. (**A**) The mRNA levels of *EZH2* in CRC tissues were compared with those in normal colon tissues using data from The Cancer Genome Atlas (TCGA) database. (**B–D**) *EZH2* expression across different stages of CRC. (**E**) Comparison of *EZH2* expression between CRC patients with complete response (CR) and those with progressive disease (PD). (**F**) Diagnostic ROC curve distinguishing CRC tissues from normal tissues based on *EZH2* expression levels. (**G-H**) mRNA and protein levels of EZH2 in normal human intestinal epithelial cells (HCM460) and CRC cell lines (HCT116, SW480, and SW48). *P<0.05, **P<0.01, ***P<0.001. CRC, colorectal cancer; EZH2, enhancer of zeste homolog 2.

*EZH2* is a critical epigenetic regulator known to be dysregulated across various cancer types, playing a pivotal role in tumorigenesis. In this study, we observed a striking up-regulation of *EZH2* in CRC tissues compared with normal colorectal tissues, as reported in The Cancer Genome Atlas (TCGA). Notably, while elevated *EZH2* expression did not correlate with the TNM stage (Figure 1A–1D), it was associated with increased chemotherapy resistance in CRC (Figure 1E). The diagnostic potential of *EZH2* was further underscored by an area under the curve (AUC) of 0.968 (Figure 1F). To corroborate the findings from TCGA, we examined *EZH2* expression in CRC cell lines (HCT116, SW480, and SW48) compared with normal colonic epithelial cells (NCM460). Consistent with the TCGA data, EZH2 expression was markedly elevated in the HCT116, SW480, and SW48 cell lines at both RNA and protein levels (Figure 1G and H). Collectively, these results suggest that heightened EZH2 expression may contribute to the pathogenesis, diagnosis, and chemotherapeutic resistance of CRC.

### *EZH2* promotes the proliferation of CRC cells *in vitro*

**Figure 2:**
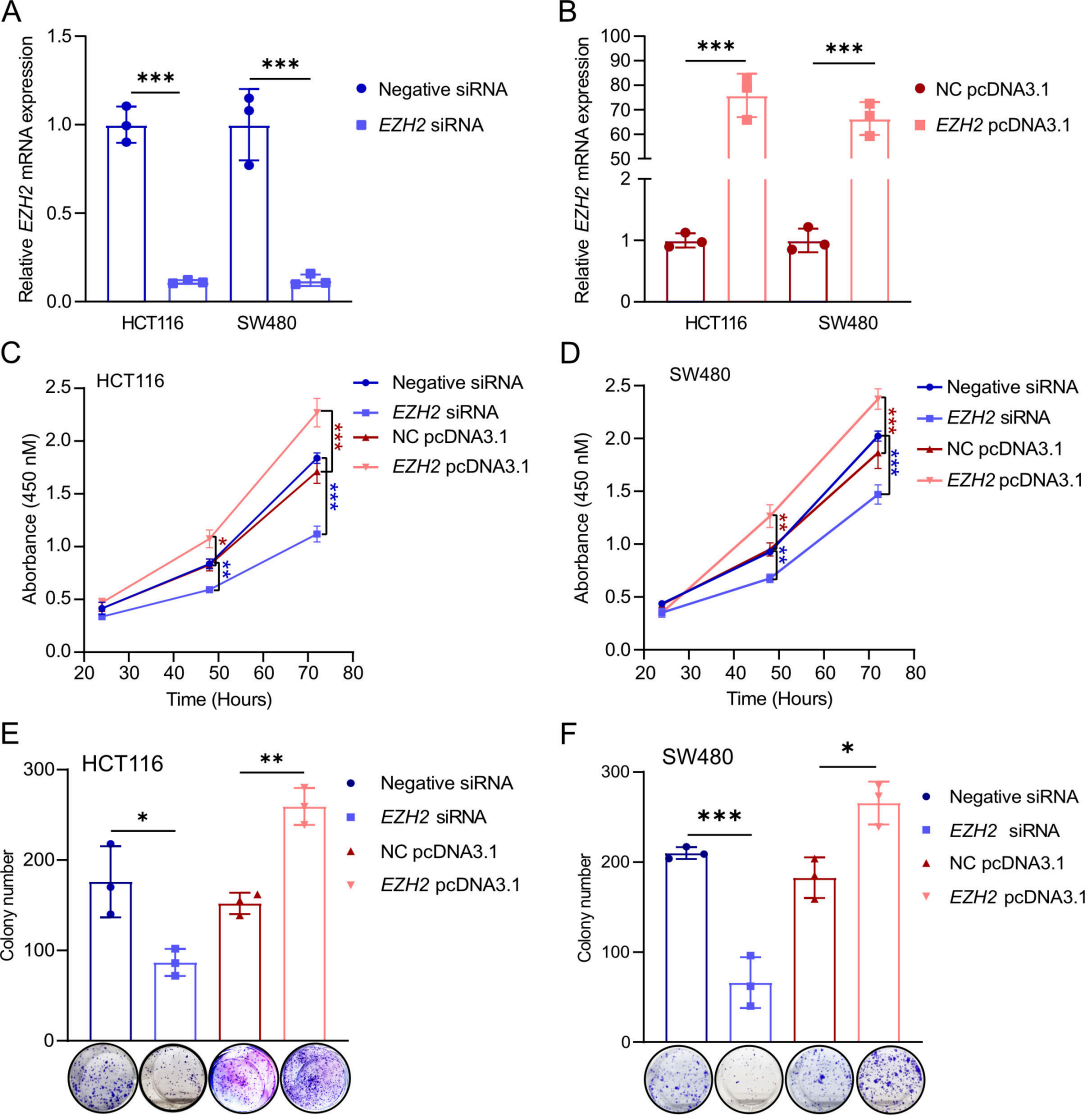
EZH2 promotes proliferation in SW480 and HCT116 cells. (**A,B**) qPCR analysis of *EZH2* mRNA levels in HCT116 and SW480 cells 48 hours after transfection with control siRNA or *EZH2* siRNA, as well as with negative control pcDNA3.1 or *EZH2* pcDNA3.1. (**C–H**) MTT assay assessing cell viability in HCT116 and SW480 cells 48 hours after *EZH2* silencing using siRNA or overexpression via *EZH2* pcDNA3.1. (**E,F**) Colony formation assay evaluating the proliferation of HCT116 and SW480 cells 48 hours after *EZH2* silencing by siRNA or ectopic overexpression of *EZH2* using pcDNA3.1. *P<0.05, **P<0.01, ***P<0.001. EZH2, enhancer of zeste homolog 2.

To investigate the functional role of *EZH2* in CRC cells, cell viability assays were performed using the SW480 and HCT116 cell lines. The results indicated that silencing *EZH2* prominently inhibited the proliferation of both SW480 and HCT116 cells, whereas overexpression of *EZH2* enhanced their proliferation (Figure 2A–2F).

### *EZH2* promotes the development of therapeutic resistance in CRC

**Figure 3:**
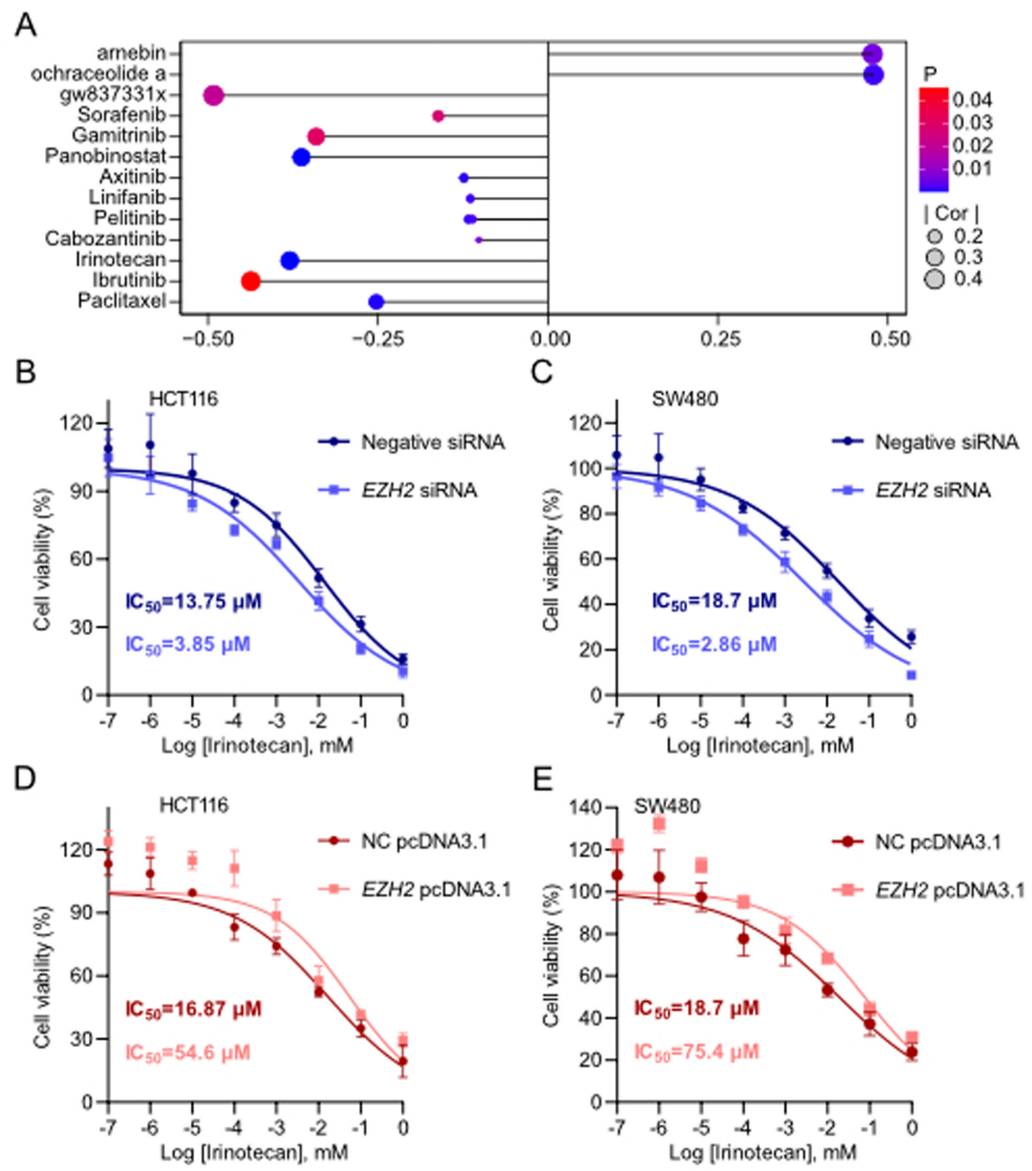
The association between EZH2 expression and drug sensitivity in CRC cells. (**A**) Analysis of the correlation between *EZH2* expression and drug sensitivity using data from the RNAactDrug database (http://bio-bigdata.hrbmu.edu.cn/RNAactDrug). (**B–E**) HCT116 and SW480 cells were treated with the indicated concentrations of irinotecan for 48 hours following *EZH2* knockdown using siRNA or overexpression via *EZH2* pcDNA3.1 for 48 hours. Cell viability was then assessed using the MTT assay. *P<0.05, **P<0.01, ***P<0.001. CRC, colorectal cancer; EZH2, enhancer of zeste homolog 2.

Subsequently, the impact of elevated *EZH2* expression on the efficacy of chemotherapeutic agents was investigated. An analysis of the RNAactDrug database (http://bio-bigdata.hrbmu.edu.cn/RNAactDrug) revealed a negative correlation between *EZH2* expression and the sensitivity to drugs such as paclitaxel and irinotecan (Figure 3A). To further investigate the role of EZH2 in modulating irinotecan efficacy, proliferation assays were conducted on HCT116 and SW480 cells with either knockdown or overexpression of EZH2. The results indicated that the IC_50_ values for irinotecan were lower in wildtype (WT) HCT116 and SW480 cells compared with those in the *EZH2* knockdown cells (Figure 3B and C). Conversely, *EZH2* overexpression increased IC_50_ values in both HCT116 and SW480 cells (Figure 3D and E).

### Irinotecan induces autophagy in HCT116 and SW480 cells by down-regulating *EZH2* expression

**Figure 4:**
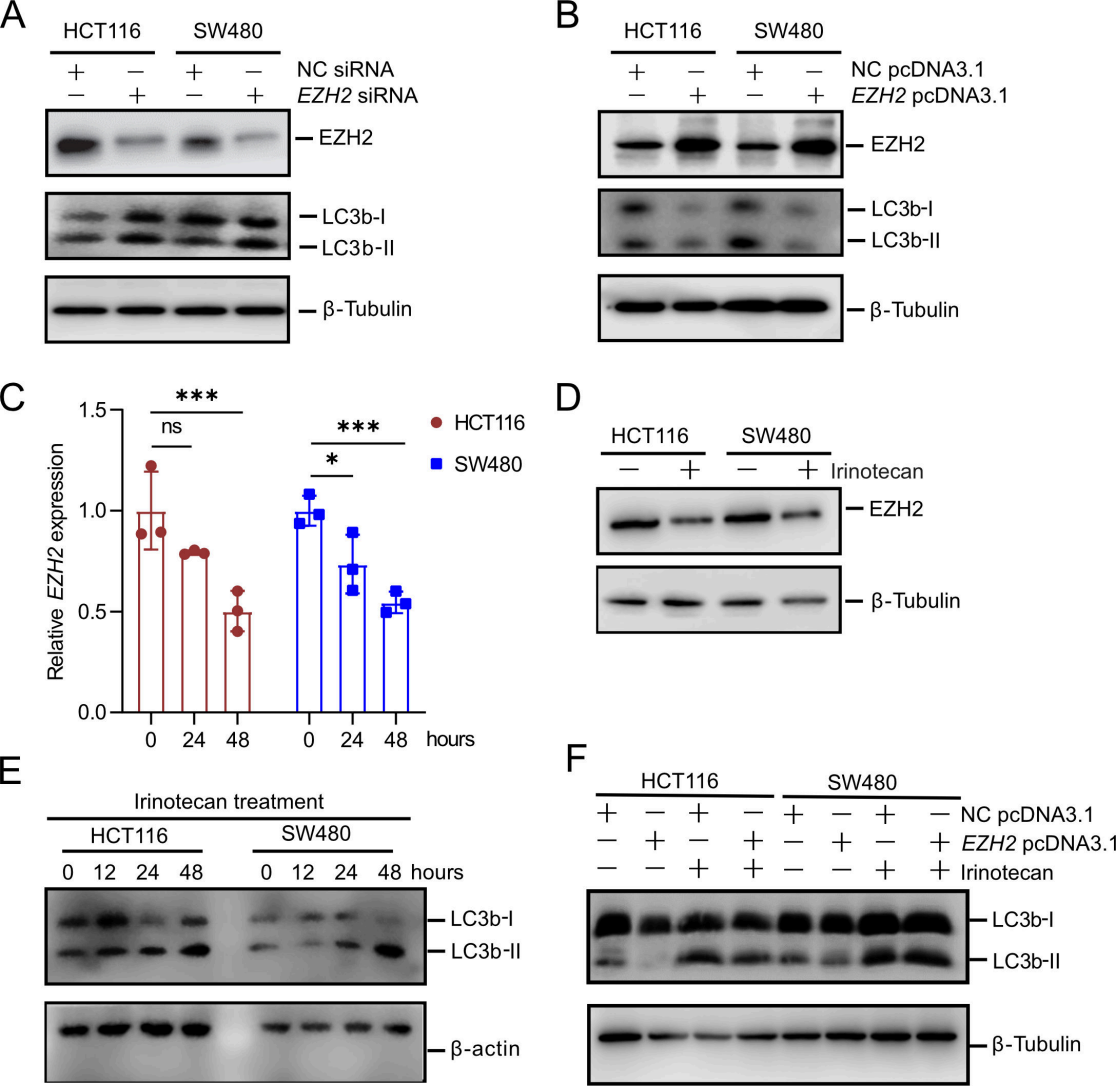
Irinotecan promotes autophagy in HCT116 and SW480 cells by down-regulating EZH2 expression. (**A,B**) Western blot analysis of indicated proteins expression in HCT116 and SW480 cells was performed 48 hours after transfection with either control siRNA or *EZH2* siRNA, as well as with negative control pcDNA3.1 or *EZH2* pcDNA3.1. (**C,D**) qPCR and Western blot analysis of EZH2 expression in HCT116 and SW480 cells were performed 48 hours after treatment with irinotecan. (**E**) Western blot analysis of LC3b-I/II expression in HCT116 and SW480 cells following 48-hour irinotecan treatment. (**F**) Western blot analysis of LC3b-I/II expression in HCT116 and SW480 cells transfected with either negative control pcDNA3.1 or *EZH2* pcDNA3.1, followed by 48 hours of irinotecan treatment. EZH2, enhancer of zeste homolog 2.

To further elucidate the mechanism by which elevated EZH2 expression contributes to chemotherapy resistance, its role in regulating autophagy was examined by assessing LC3bI/II levels. The results illustrated that *EZH2* knockdown in HCT116 and SW480 cells led to an increase in LC3b-II expression, indicating enhanced autophagy (Figure 4A). Conversely, *EZH2* overexpression resulted in a decrease in LC3bII levels, suggesting reduced autophagy inhibition (Figure 4B).

To confirm that irinotecan promotes autophagy in CRC cells by inhibiting EZH2 expression, we investigated its effects on both mRNA and protein levels of EZH2. The results showed that irinotecan remarkably reduced EZH2 expression in CRC cells (Figure 4C and D). Following irinotecan treatment of HCT116 and SW480 cells, a pronounced increase in LC3b-II expression was observed at 48 hours (Figure 4E). Furthermore, *EZH2* overexpression partially reversed the irinotecan-induced increase in LC3b-II expression in these cells (Figure 4F). These findings suggest that irinotecan-mediated suppression of EZH2 is crucial for inducing autophagy in CRC cells.

### Elevated *EZH2* expression in CRC cells contributes to irinotecan resistance

**Figure 5:**
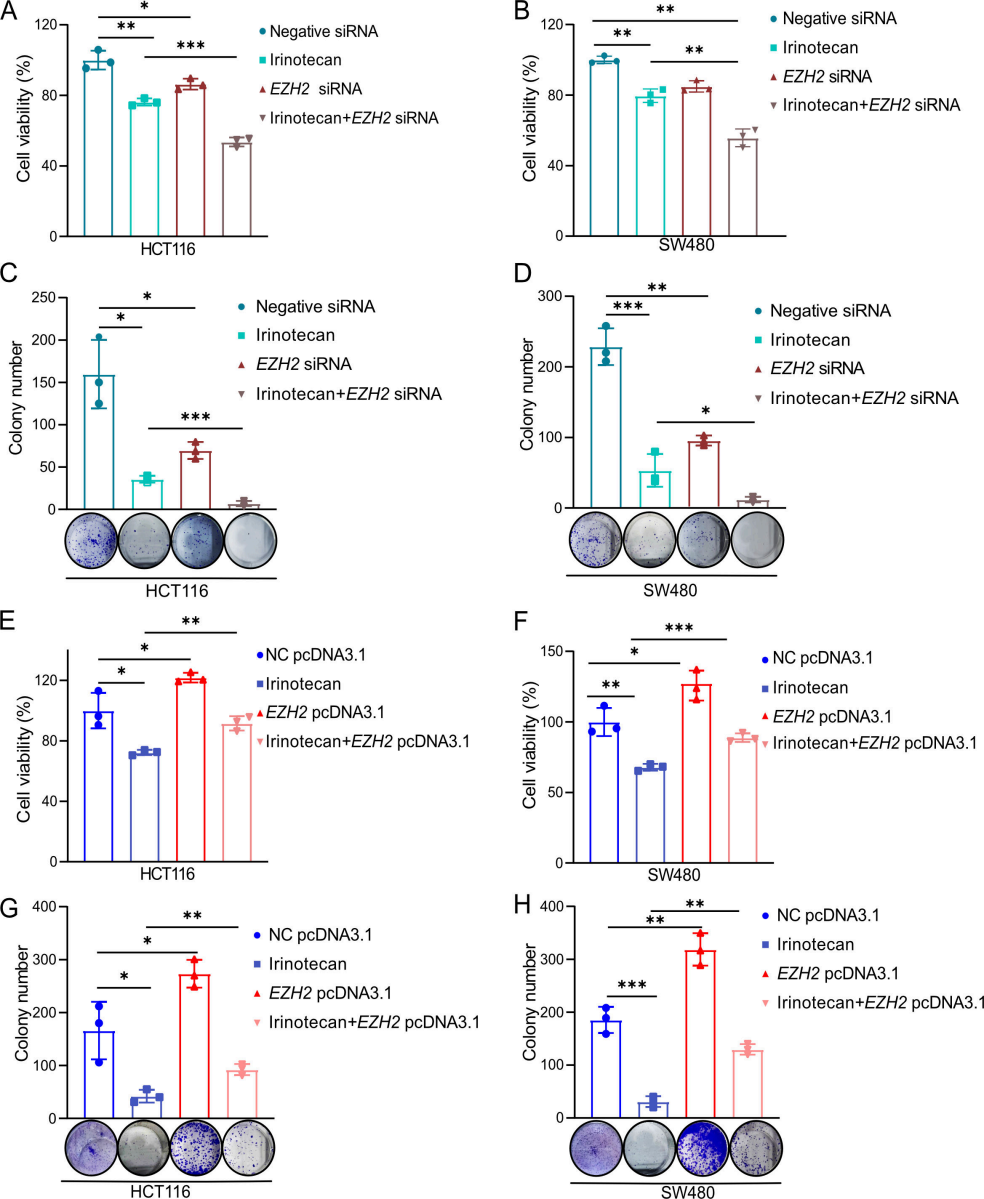
High EZH2 expression confers irinotecan resistance in CRC cells. (**A,B**) Cell viability of HCT116 and SW480 cells transfected with either control siRNA or *EZH2* siRNA, followed by irinotecan treatment for 48 hours, was assessed using the MTT assay. (**C,D**) Colony formation assay was used to evaluate cell proliferation in HCT116 and SW480 cells transfected with either control siRNA or *EZH2* siRNA followed by treatment with irinotecan for 48 hours. (**E–H**) MTT and colony formation assays were used to analyze cell viability and proliferation capacity in HCT116 and SW480 cells transfected with either control or *EZH2* siRNA, followed by irinotecan treatment for 48 hours. CRC, colorectal cancer; EZH2, enhancer of zeste homolog 2.

To investigate whether elevated EZH2 expression in CRC cells contributes to irinotecan resistance by inhibiting autophagy, we examined the impact of EZH2 modulation on cell viability and proliferation following irinotecan treatment. Using either *EZH2* knockdown or *EZH2* overexpression, we analyzed the effect of irinotecan on HCT116 and SW480 cells. Irinotecan obviously reduced both proliferation and viability in these cells. Moreover, *EZH2* knockdown via siRNA enhanced the inhibitory effect of irinotecan on cell proliferation (Figure 5A–5D). In contrast, ectopic overexpression of *EZH2* attenuated the ability of irinotecan to suppress proliferation in HCT116 and SW480 cells (Figure 5E–5H). These findings highlight the pivotal role of EZH2 in mediating resistance to irinotecan chemotherapy in CRC.

### *EZH2* induces NRP1 expression by binding to the NRP1 promoter region

**Figure 6:**
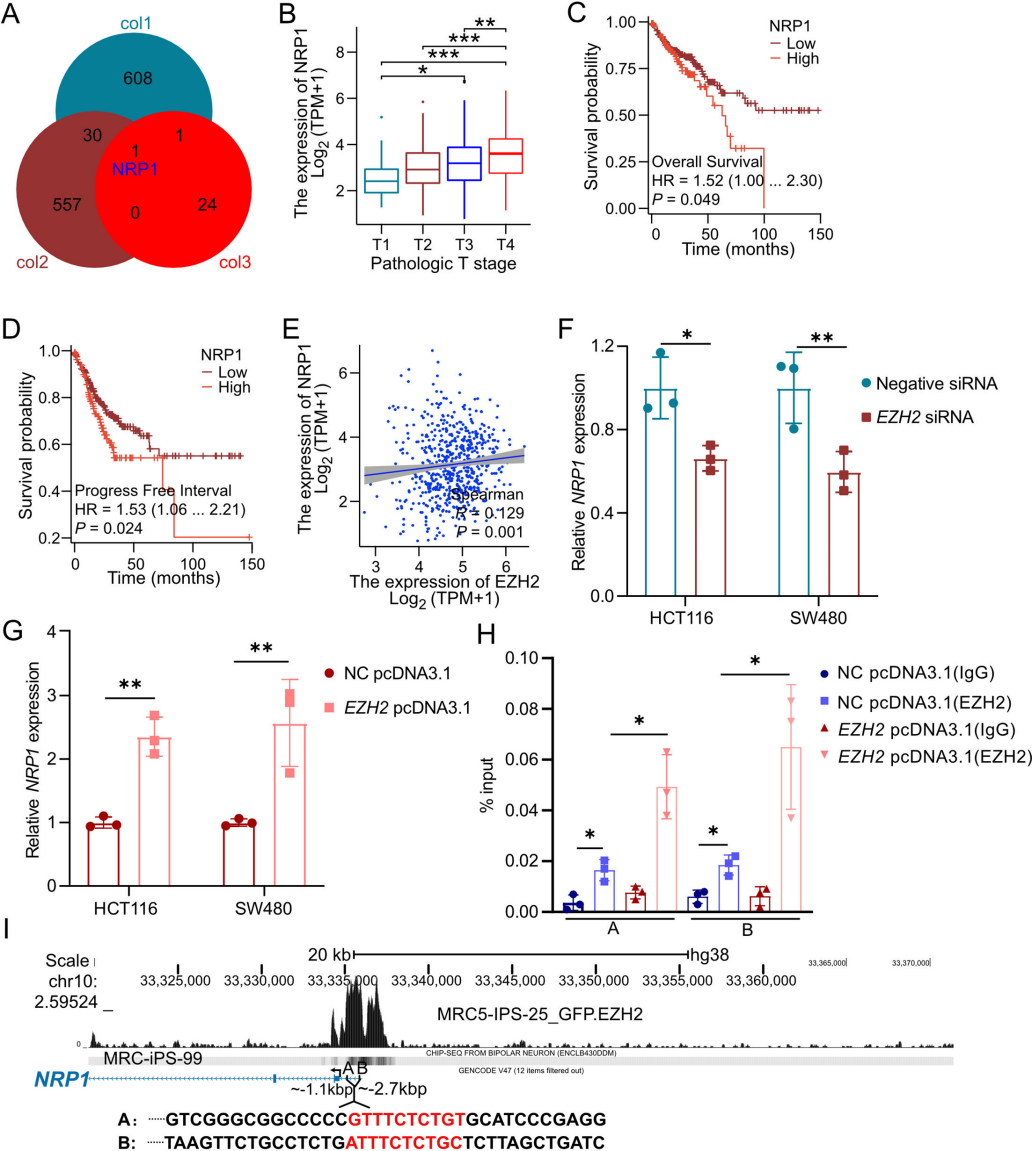
EZH2 regulates NRP1 expression by binding to the NRP1 promoter region. (**A**) Venn diagram showing the overlap of differentially expressed genes in cancer cells after *EZH2* depletion or silencing, as identified in datasets GSE18150, GSE73294, and GSE115010. (**B**) *NRP1* expression across different T stages in colon cancer patients from the TCGA dataset. (**C,D**) Progression-free interval (PFI) and overall survival (OS) analyses of CRC patients based on NRP1 expression levels from the TCGA dataset. (**E**) Spearman correlation analysis between NRP1 and EZH2 expression in CRC patients from the TCGA dataset. (**F,G**) NRP1 expression in HCT116 and SW480 cells 48 hours after the indicated treatments. (**H**) qCHIP assay for EZH2 binding to the NRP1 A and B promoters in HCT116b cells. (**I)** Screenshot from the UCSC Genome Browser showing ChIP-seq peaks for NRP1 and EZH2 on human chromosome 10. CRC, colorectal cancer; EZH2, enhancer of zeste homolog 2; NRP1, neuropilin-1.

To elucidate how EZH2 regulates autophagy and contributes to irinotecan resistance in CRC, we analyzed its role in modulating NRP1 expression. Knockdown or depletion of *EZH2* in tumor cells significantly enhanced the inhibitory effects of paclitaxel, docetaxel, and cisplatin on tumor cells, leading to a striking reduction in *NRP1* levels (Figure 6A). Clinical analysis using the TCGA dataset revealed that NRP1 expression was significantly higher in CRC patients with T4-stage tumors compared with those with T1-stage tumors (Figure 6B). Additionally, CRC patients with elevated *NRP1* expression exhibited shorter overall survival (Figure 6C–D). Furthermore, a positive correlation between *EZH2* and *NRP1* expression was observed (Figure 6E).

To further validate this regulatory relationship, we observed that knockdown *EZH2* reduced *NRP1* levels, whereas *EZH2* overexpression significantly increased *NRP1* expression in CRC cells (Figure 6F and G). Mechanistically, we discovered that EZH2 directly binds to the promoter region of *NRP1*. EZH2 was enriched at the *NRP1* promoter region (Figure 6H and I). Collectively, these findings suggested that EZH2 regulated the transcription of *NRP1* by binding to its promoter region in CRC cells.

### The inhibition of autophagy mediated by *EZH2* contributes to irinotecan resistance, partially dependent on NRP1

**Figure 7:**
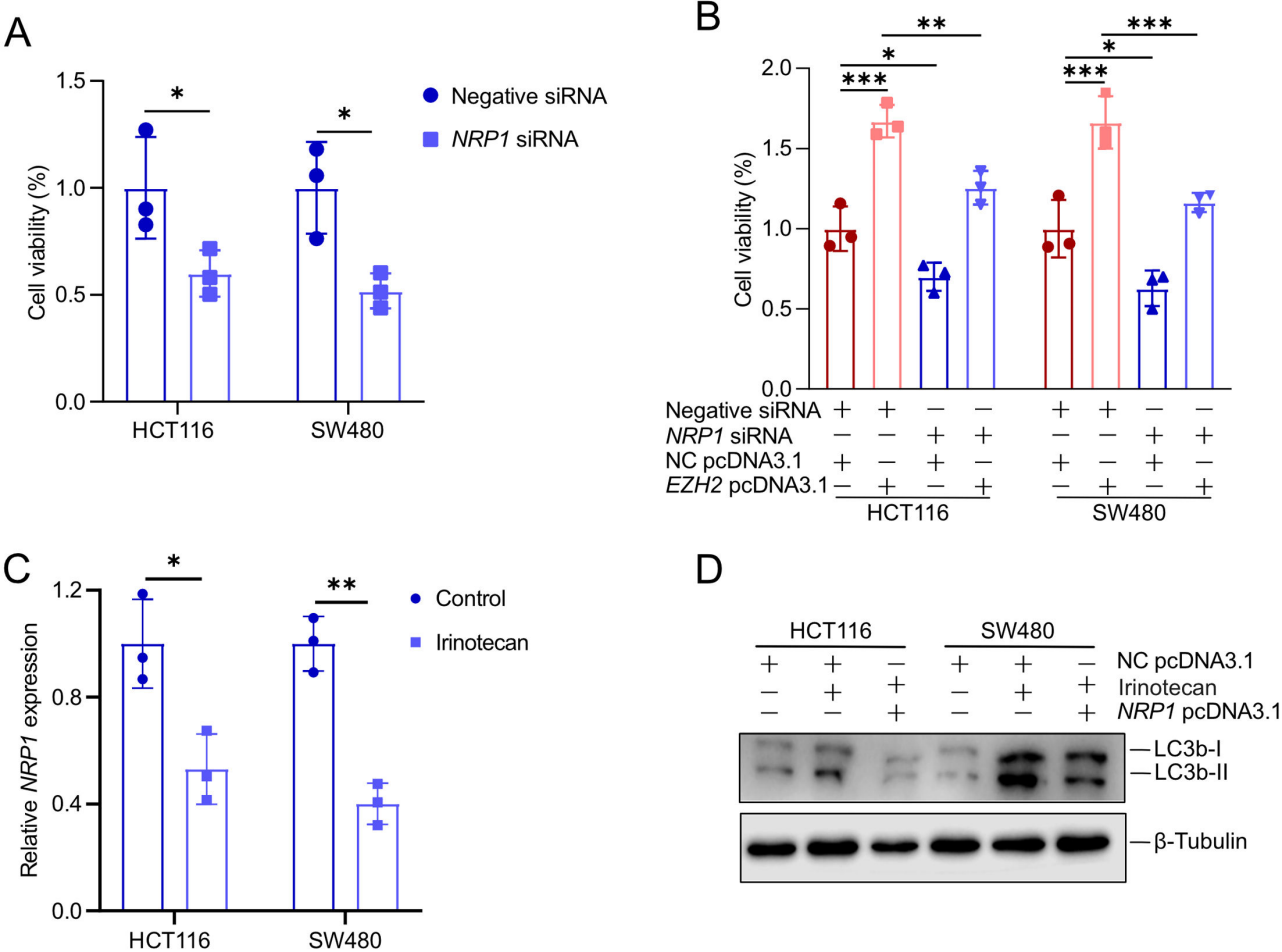
EZH2-mediated inhibition of autophagy contributes to irinotecan resistance, partially through NRP1. (**A-B**) MTT assay evaluating cell viability in HCT116 and SW480 cells after 48 hours of the indicated treatments (**C**) qPCR analysis of *NRP1* expression in HCT116 and SW480 cells was performed 48 hours after treatment with irinotecan. (**D**) Western blot analysis of LC3b-I/II expression in HCT116 and SW480 cells after 48 hours of the indicated treatments. EZH2, enhancer of zeste homolog 2; NRP1, neuropilin-1.

Our findings indicated that *NRP1* knockdown significantly suppressed the growth of HCT116 and SW480 cells (Figure 7A). Furthermore, *NRP1* knockdown partially reversed the effects of *EZH2* overexpression on cell proliferation (Figure 7B). Irinotecan reduced *NRP1* expression and increased LC3b-II expression in HCT116 and SW480 cells (Figure 7C and D). In contrast, ectopic overexpression of NRP1 attenuates the irinotecan-induced increase in LC3b-II expression in HCT116 and SW480 cells (Figure 7D). These results suggest that EZH2 promotes CRC cell proliferation and suppresses autophagy by regulating NRP1 expression, thereby contributing to irinotecan resistance.

## Discussion

Our study aimed to explore the mechanism by which EZH2 contributes to chemotherapeutic resistance in CRC by inhibiting autophagy. Specifically, elevated levels of EZH2 in CRC tissues and cells promote tumor cell proliferation and chemoresistance to irinotecan by suppressing autophagy. Mechanistically, EZH2 directly binds to the *NRP1* promoter, up-regulating its expression and further enhancing chemoresistance in CRC cells. Depletion of either *EZH2* or *NRP1* sensitizes CRC cells to irinotecan, suggesting that the EZH2-NRP1 axis plays a critical role in modulating therapeutic resistance.

EZH2, a conserved histone lysine methyltransferase [20], is frequently overexpressed or mutated in a wide range of cancers, where it promotes tumor initiation, progression, and poor clinical outcomes [21–24]. Corroborating with these previous findings, our study demonstrated that EZH2 significantly overexpressed in CRC tissues and cell lines. The high diagnostic accuracy of *EZH2*, reflected by the AUC of 0.968, underscores its potential as a biomarker for CRC diagnosis and prognosis. EZH2 promoted the proliferation of CRC cells, as evidenced by reduced cell viability and colony formation following *EZH2* depletion and enhanced proliferation upon *EZH2* overexpression. EZH2 exerts oncogenic effects through various mechanisms, including phosphorylation-mediated modulation of its function [25], its role in the PRC2 complex with EED and SUZ12 to drive histone methylation [26,27], and its promotion of metabolic reprogramming by enhancing tricarboxylic acid cycle activity [28]. Additionally, previous studies have shown that EZH2 activates the PI3K/Akt pathway, contributing to oxaliplatin resistance [29]. Although we observed no strong correlation between *EZH2* expression and TNM stage, our data indicated that elevated EZH2 levels were associated with increased resistance to chemotherapy, particularly irinotecan.

In our study, the mechanism through which EZH2 exerts its pro-tumorigenic effects is derived from its chemoresistance, which is imculpated to involve the inhibition of autophagy, which is a primary cellular degradation process responsible for removing damaged macromolecules and organelles [11]. Our results indicated that *EZH2* depletion induced autophagy in CRC cells, as indicated by increased LC3bII expression, whereas *EZH2* overexpression suppressed this process. Notably, irinotecan induced autophagy by down-regulating *EZH2* expression. However, this effect was partially reversed by ectopic *EZH2* expression, reinforcing the notion that EZH2 plays a central role in modulating autophagy in response to chemotherapy. These findings suggest that EZH2-mediated autophagy inhibition provides a survival advantage to CRC cells, enabling them to withstand chemotherapeutic stress.

Previous studies have illustrated the regulation of autophagy by EZH2 through an mTOR-dependent pathway [11], which can be suppressed by NRP1 complex [30]. Given that NRP1 is involved in tumor progression across various cancer types [31–36], our study identified synchronous changes and positive correlation between EZH2 and NRP1 in CRC. Additionally, we demonstrated that *NRP1* is a downstream target of EZH2, providing evidence that EZH2 directly binds to the *NRP1* promoter and induces its expression. This drives tumor progression and resistance to irinotecan, with *NRP1* knockdown partially reversing the effects of EZH2 overexpression on both cell proliferation and autophagy suppression.

Collectively, our findings suggest that EZH2 and NRP1 co-operatively contribute to CRC progression and chemoresistance through the modulation of autophagy. Targeting the EZH2-NRP1 axis may represent a promising therapeutic strategy to enhance the efficacy of chemotherapy in CRC patients.

## Method

### Cell culture

Human normal colon epithelial cells (NCM460) and human CRC cell lines (HCT116, SW48, and SW480), originally obtained from the American Type Culture Collection, were used in this study. NCM460 cells were cultured in M3:10A™ medium, HCT116 cells in McCoy’s 5A medium, and SW48 and SW480 cells in Dulbecco’s modified Eagle medium. All cells were maintained in a humidified atmosphere at 37°C with 5% CO₂. The culture media were supplemented with 10% fetal bovine serum, 100 U/ml penicillin, and 100 µg/ml streptomycin. Irinotecan hydrochloride (#I1406, Sigma) was prepared as a 10-mM stock solution in DMSO.

### RNA isolation and quantitative real-time polymerase chain reaction (qPCR) analysis

Total RNA was isolated and purified using the High Pure RNA Isolation Kit (Roche), according to the manufacturer’s instructions. Complementary DNA (cDNA) was synthesized from 1 µg of RNA per sample using the Verso cDNA Synthesis Kit (Thermo Fisher Scientific, Waltham, MA). qPCR was performed using Fast SYBR™ Green Master Mix (Applied Biosystems, Foster City, CA). The _ΔΔ_Ct method was used for relative gene expression analysis, with β-actin serving as the internal control for normalization. The sequences of qPCR primers are provided in Supplementary Table S1.

### MTT assay

HCT116 and SW480 cells were seeded into 96-well plates at a density of 3000 cells per well. Cells were transfected with the indicated siRNAs, *EZH2* pcDNA3.1, or vector controls. After 24 hours, the cells were treated with the specified concentrations of irinotecan for 48 hours. Following the treatment, 10 µl of MTT solution (0.5 mg/ml) was added to each well and incubated for 4 hours. The culture medium was then carefully removed, and 100 µl of DMSO was added to dissolve the formazan crystals. After mixing the plate, absorbance was measured at 570 nm using the Varioskan system (Thermo Fisher).

### Western blot analysis

Cells were lysed in RIPA buffer supplemented with protease inhibitors (Roche) and PhosSTOP™ phosphatase inhibitor tablets (Roche). Lysates were sonicated for 5 seconds and centrifuged at 15,000 rpm for 20 minutes at 4°C. The supernatant was collected, and protein concentrations were quantified using the Pierce™ BCA Protein Assay Kit (Thermo Fisher Scientific). For each sample, 40 µg of protein was separated by 10% or 12% sodium dodecyl sulfate–polyacrylamide gel. Proteins were transferred onto immobilon PVDF membranes (Millipore) following standard protocols (Bio-Rad Laboratories). Imaging was performed using the ECL (Millipore) and visualized with LI-COR Odyssey FC imaging system (Bad Homburg, Germany). The primary antibodies were shown as follows: anti-β-tubulin (2146 cell, signaling), anti-EZH2 (5246, cell signaling), and anti-LC3b-I/II (3868, cell signaling).

### Colony formation assay

Cells were seeded in six-well plates and transfected with the indicated siRNA, *EZH2* pcDNA3.1, or vector for 24 hours before treatment with irinotecan for 48 hours. Cells were resuspended and 2000 cells per well were re-seeded into new six-well plates. Colony formation was assessed after two weeks of incubation. Colonies were imaged using a digital camera and quantified with ImageJ software.

### Chromatin immunoprecipitation

HCT116 cells were transfected with *EZH2* pcDNA3.1 for 48 hours to activate EZH2. ChIP experiments were performed using the PureBinding® Chromatin Immunoprecipitation Kit. The sequences of qChIP primers are provided in Supplementary Table S2.

### Bioinformatics analysis

Gene expression levels were normalized using housekeeping genes, and the geometric mean of ranks (rank product) was calculated for per-sample gene expression. For differential gene expression analysis of unpaired samples, data from UCSC XENA’s unified processing of TCGA and GTEx RNA-seq data (in TPM format, processed by Toil) were used, comprising 51 normal human colorectal tissue samples and 179 CRC tissue samples. RNA-seq data from the TCGA CRC project (level 3, HTSeq-FPKM format) were utilized for correlation analysis of potentially related molecules. FPKM values were converted to TPM and log₂-transformed to allow for the comparison of expression levels. The Mann–Whitney *U* test (Wilcoxon rank-sum test) and Spearman’s correlation analysis were conducted using the ggplot2 (version 3.3.3) R package.

### Statistical analyses

Data were presented as mean ± SD from triplicate experiments and analyzed using GraphPad Prism 9 Software (GraphPad, U.S.A.), which was used for all graphical representations. Statistical comparisons between groups were performed using Student’s *t*-test, while the Wilcoxon signed-rank test was applied to account for unequal variances between groups. A *P* value of <0.05 was considered the threshold for statistical significance.

## Highlights

1. Elevated enhancer of zeste homolog 2 (EZH2) expression in colorectal cancer (CRC is associated with chemoresistance).
2. EZH2 promotes CRC cell proliferation and chemoresistance by suppressing autophagy.
3. EZH2 directly regulates neuropilin-1 (NRP1) expression, driving tumor growth and resistance to irinotecan.
4. *NRP1* knockdown mitigates EZH2-mediated chemoresistance and partially restoring autophagy.

## Supplementary material

Online supplementary tables

## Data Availability

All authors confirm that the data supporting the findings of this study are available within the article. The datasets used and/or analyzed during the current study are available from the corresponding author upon reasonable request.

## Abbreviations

cDNA: complementary DNA
CRC: colorectal cancer
EZH2: enhancer of zeste homolog 2
NRP1: neuropilin-1
TGCA: The Cancer Genome Atlas
