# Supplementary material for: EZH2 promotes chemoresistance in colorectal cancer by inhibiting autophagy through NRP1 suppression
Source: Biochem J. 2025 May 14;482(10):569–81. doi: 10.1042/BCJ20240607 (PMC12203955; doi:10.1042/BCJ20240607)
Supplement: Online supplementary tables [file BCJ-482-10-BCJ20240607-s001.doc]

Table S1： Oligonucleotides used for qPCR.

| mRNA | Forward (5’-3’) | Reverse (5’-3’) |
| --- | --- | --- |
| *EZH2* | TGGTGAATGCCCTTGGTCAA | AGTTCTTCTGCTGTGCCCTT |
| *β-actin* | TGACATTAAGGAGAAGCTGTGCTAC | GAGTTGAAGGTAGTTTCGTGGATG |
| *NRP1* | TCCAGCGGCTCACAAAGAAT | ACGTCTAGCAATGTGGGAAGG |

Table S2: Oligonucleotides used for qChIP.

| mRNA | Forward (5’-3’) | Reverse (5’-3’) |
| --- | --- | --- |
| *NRP1*  (site A) | GAGGGGTGTGGAAATCAGGG | CTGTGGGAAGTGGGGGTG |
| *NRP1*  (site B) | CCAGCTTTCTCCAACCAGTGA | GTGCGGTAGGGCTCCATTAC |
